# Turning unprofessional behaviors around using Holmes' reflection approach: a randomized controlled study

**DOI:** 10.18502/jmehm.v13i12.4388

**Published:** 2020-09-16

**Authors:** Leila Naeimi, Fariba Asghari, Saharnaz Nedjat, Azim Mirzazadeh, Mahsa Abbaszadeh, Ali Reza Sima, Sara Mortaz Hejri

**Affiliations:** 1 *Researcher, Department of Medical Education, School of Medicine, Tehran University of Medical Sciences, Tehran, Iran; Medical Education Development Center, Zanjan University of Medical Sciences, Zanjan, Iran.*; 2 *Associate Professor, Medical Ethics and History of Medicine Research Center, Tehran University of Medical Sciences, Tehran, Iran.*; 3 *Professor, Department of Epidemiology and Biostatistics, School of Public Health, Tehran University of Medical Sciences, Tehran, Iran.*; 4 *Associate Professor, Department of Medical Education, Health Professions Education Research Center, Tehran University of Medical Sciences, Tehran, Iran.*; 5 *Assistant Professor, Department of Internal Medicine, Imam Khomeini Hospital Complex, Tehran University of Medical Sciences, Tehran, Iran.*; 6 *Assistant Professor, Digestive Disease Research Center, Digestive Disease Research Institute, Shariati Hospital, Tehran University of Medical Sciences, Tehran, Iran.*; 7 *Assistant Professor, Department of Medical Education, Educational Development Center, Tehran University of Medical Sciences, Tehran, Iran.*

**Keywords:** Professionalism, Holmes' approach, Unprofessional behavior, Medical students, Reflection.

## Abstract

Many medical schools around the world have included professionalism training in their formal curriculum. However, these efforts may not be adequate; given the exposure of students to unprofessional behaviors in the clinical settings. In the present study, we aimed to design, implement, and evaluate a longitudinal program to improve professionalism among medical students upon their transition to clinical settings. A total of 75 medical students were enrolled in the study and randomly assigned to two groups. The control group did not receive any training, while for the intervention group; a 10-hour program through 16 weeks was organized based on the Holmes' reflection approach. The effectiveness of the program was evaluated by measuring three outcomes in both groups. Data analysis was performed using paired t-test and Multiple Linear Regression. Scores of judgment of professionalism increased in the intervention group (from 7.56 to 10.17; *P*< 0.001), while there was no significant improvement in the control group’s scores. Students' attitudes towards professionalism and their professional behaviors did not change significantly.

Based on our findings, the Holmes reflection approach helps students improve their cognitive base of professionalism. Long-term follow-up and further qualitative studies will help us better understand the effects of this approach on other desirable outcomes.

## Introduction

Professionalism, one of the six core competencies proposed by the Accreditation Council of Graduate Medical Education (1), has become increasingly important for the medical community, as well as the public (2,3). The results of previous studies showed that demonstration of professional behaviors by physicians increases patients' satisfaction and trust, help patients adhere to their therapeutic plans, and reduce medical litigations (4-6). Hence, medical students should be trained to establish professional behaviors and provide high-quality patient-centered care in the future (1).

The development and maintenance of professionalism are one of the challenging issues in medical education. It is important to explicitly teach professionalism and actively reinforce the desired values, throughout both the undergraduate and postgraduate curricula (7). Apart from role modeling which seems to be the most effective technique for developing professionalism and besides didactic sessions which are regularly used to provide the cognitive base of professionalism, experiential learning, reflective practice, and methods of critical and guided reflection are strongly recommended for endorsing professionalism (8-10).

Reflection is described as a core element of clinical competence and a fundamental element in teaching professionalism (11). Physicians often have to resolve complex problems with no predefined solutions. Hence, exposing students to the complexities of their professional life is essential and guided reflection can help maximize their learning (12). Despite its variability, reflection models are not usually integrated into medical education programs due to time constraints and feasibility issues (11). In this study, we decided to use the Holmes' reflection approach, which suggests a set of reflective competencies that are helpful, especially for medical students who have been exposed to unprofessional behaviors, during their transition from pre-clinical period to clinical settings (13, 14). In this approach, similar to the Schön's model, it is assumed that reflective practice could take place when a person is faced with a real problem that has no simple solution. While the Schön's model could be applied for all individuals in any circumstances, the Holmes' approach is specifically designed for medical students who have been exposed to unprofessional behaviors in the clinical settings. This model is based on the observation of events in real situations: It focuses on unprofessional behaviors, which are inevitable parts of the hidden curriculum at the workplace; It targets students who are new in their clinical role, and therefore, are more likely to be sensitive toward the professional lapses that occur around them; and it facilitates reflection using certain four-step strategies, namely, priming, noticing, reflecting, and choosing. 

There is little evidence concerning the application and effectiveness of the Holmes' approach. Seen from a broader perspective, research in the field of medical professionalism is mostly composed of descriptive reports or qualitative studies, most of which have discussed the importance of professionalism or have elaborated on different methods of teaching professionalism (8, 9, 15). A limited number of studies have focused on the impact of teaching methods and have indeed evaluated the results of educational interventions on professionalism (11, 16-18). These studies have shed light on this area; however, to the best of our knowledge, they have failed to evaluate the impact of their program on the students' knowledge base or actual professional behaviors (15-16), nor have used a particular theoretical framework for reflection (16), or have not recruited an identical control group to provide a basis for more valid comparisons (16-18). 

In this study, we tried to provide robust evidence by evaluating the educational outcomes of our longitudinal program at different levels of reaction, learning, and behavior using the Holmes' reflection approach. We chose clerks (fourth year medical students) because noticing and observing unexpected behaviors, which are in contrast with the ethical values learned in the formal curriculum, more likely occurs at the beginning of a new period of training.

## Method

***Study design***

**Figure 1:**
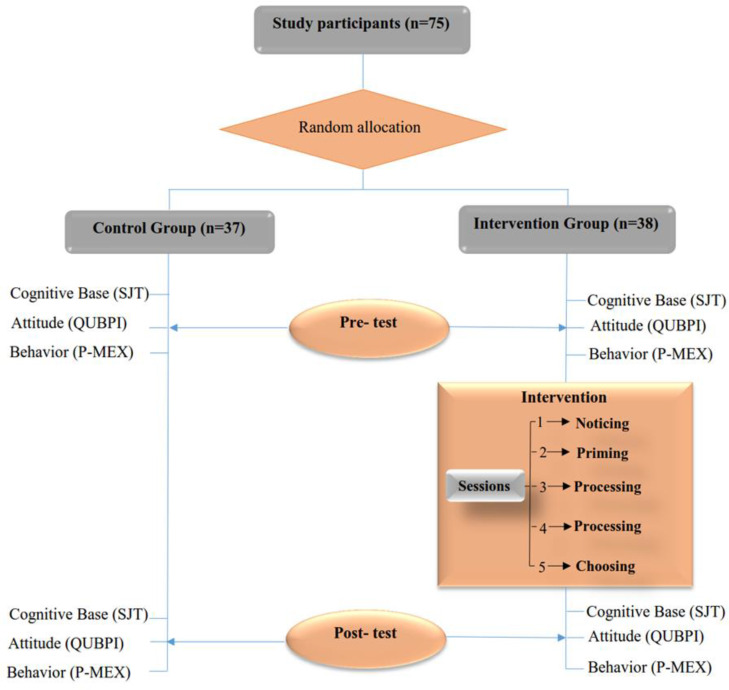
The study design and evaluated outcomes

This pre-test post-test randomized controlled study was conducted in 2017-2018 on medical students. TUMS medical students are mainly admitted to the university from high school and the total duration of their training, including the internship, is seven years. The preclinical period is an integrated organ system-based curriculum that continues for three and a half years. These medical students have to follow two unit course in ethics in the pre-clinical period. While these students have an early clinical exposure during the pre-clinical period, their first clinical experience at the patient's bedside starts in the fourth year when they are assigned to the university-affiliated hospitals for their clerkship. The internal medicine clerkship was selected for this study, as it is the longest rotation (16 weeks) and the starting point of clinical practice for many students. In the briefing session at the beginning of the internal medicine clerkship, the study objectives and procedures were described for all students, and those who were willing to participate were enrolled in the study. For data analysis, we excluded students who were absent for two or more sessions as well as those who failed to complete the evaluations. A sample size of 23 was estimated for each group as a prior; however, concerning the probability of dropouts, we considered a larger sample size. Students were randomly assigned to the control and intervention groups. The control group did not receive any training, while the intervention group participated in five sessions. To minimize the risk of contamination, participants were asked not to share program details with friends and classmates of the control group (Figure 1).

***Design and delivery of the educational intervention***

The research team met regularly to discuss the details of the design and structure of the longitudinal program based on the Holmes’ approach. They made decisions on the schedule, teaching methods, content, and implementation of the intervention.

We finally decided to organize the whole program as five 2-hour sessions within 16 weeks. With regard to the teaching methods, we decided to use the small group technique. Hence, we randomly allocated the students into six small groups (five groups of six and one group of eight people). As we needed facilitators to coordinate discussions in small groups, we invited a number of medical interns who were collaborating with the Educational Development Office at the Medical School. These medical interns were previously trained to play the role of facilitators; yet, before the beginning of the program, we oriented them about our study objectives, the intervention procedure, and the purpose of the sessions. We also provided them with a written guideline. The facilitators were instructed to use their own experience to encourage discussions in the small groups. With regard to the content of the program, we decided to apply the Holmes' steps in five sessions:

In the first session, the program was introduced, and the importance and necessity of teaching professionalism were discussed interactively. A notebook was given to the students to record daily diaries immediately to prevent contamination of the memory. Then, the first step of Holmes' reflection approach, priming, was implemented. For this purpose, some examples of how medical students in a clinical setting might yield to external pressures to engage in inappropriate behaviors were presented. The facilitators shared their experience in this area and encouraged the students to participate in the discussion. The students were asked to pay attention to unprofessional behaviors that might occur in upcoming days during their rotation and to document their daily memories in their clinical notebooks. In the second session, according to the second step of Holmes' reflection approach, noticing, the students described the ethical issues that they were exposed to. They were encouraged to share their notes of unprofessional behaviors, to express the emotion that they had felt at those moments, and to discuss their experiences. In the third session, the third step of Holmes' reflection approach, processing, was implemented. The objective of this session was to help students develop feasible action plans to deal with similar situations in the future. In the fourth session, we repeated the processing step, so that all students would have the opportunity to reflect deeply and critically on their observations and to receive feedback. In the last session, the fourth step, choosing, was implemented. The objectives of this session were to support students in identifying and adopting behaviors aligned with the profession's values and to avoid misconducts; help students apply their action plans (an outcome of steps 3) in a way that it reinforces their progress without alienating them from their peers or seniors, and show students how to politely disagree with others. In this session, a sample of poor reflections was compared with some good reflections, and group discussions were conducted. Each facilitator was assigned to a small group and encouraged students to reflect. The facilitators provided feedback about the students' reflection and helped them distinguish a poor reflection from a good one, using a guideline (19). 

***Investigation of outcome***

**Figure 2:**
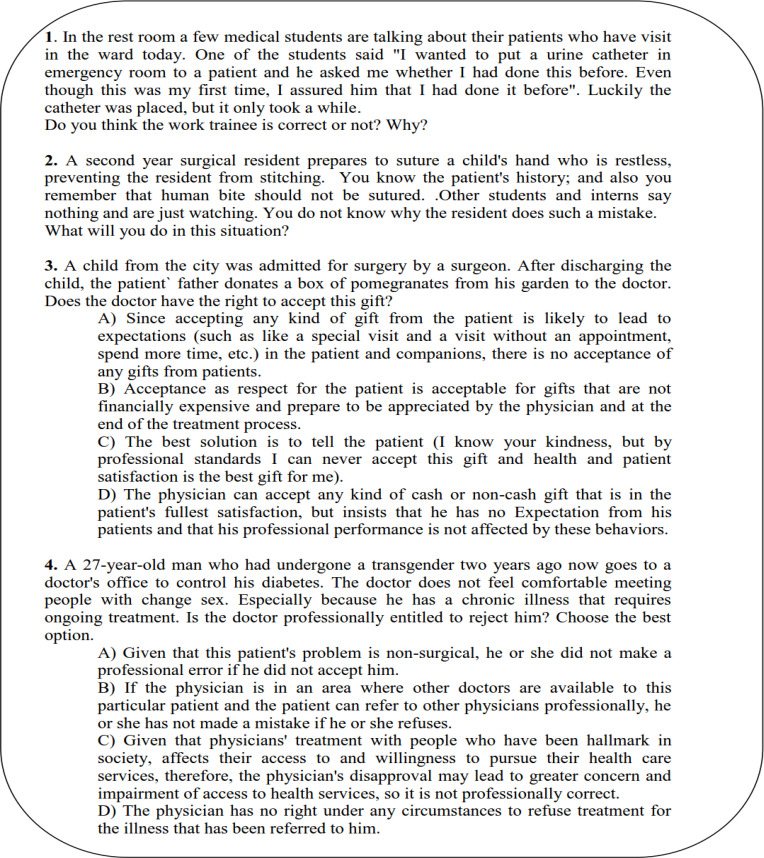
Questions of Situational Judgment Test for assessing students' professionalism cognitive base

The assessment of outcomes was carried out at the beginning of the study and one month after the program, for both intervention and control groups. We used several tools to determine the effectiveness of the program:

We evaluated the cognitive base of professionalism (level 2A of Kirkpatrick's framework) using a Situational Judgment Test (SJT), consisting of 11 clinical scenarios (seven constructed-response questions and four selected-response questions). Some examples of this test are presented in figure 2. The test was designed and scored by a panel of experts, based on medical professionalism guidelines. The total score of this test ranged from 0 to 20.

The students' attitude toward professionalism (level 2B of Kirkpatrick's framework (20)) was assessed using the modified version of Queen’s University Belfast Professionalism Index (QUBPI) inventory. The original version of this questionnaire contains two sections. The first section, with 20 items, evaluates the person’s attitude towards professional behaviors and commitment, and the second part, with 19 items, evaluates the frequency of exposure to unprofessional behaviors (21). In this study, only the first part of this tool was used. This part consists of two sub-sections, to be rated on a five-point Likert scale (completely disagree= 5; completely agree= 1). We translated and validated the questionnaire according to the guideline developed by Sousa and Rojjanasrirat (22). Concerning content validity, which was evaluated by the help of faculty members and medical students, three questions (1, 10, and 20) were removed. Therefore, the score of the Persian version ranged between 17 and 80. Moreover, the reliability of this tool was found to be desirable, based on the results of the Intraclass Correlation Coefficient and Cronach's alpha measurements (≥ 0.70). 

Moreover, the students' professional behaviors were evaluated (level 3 of Kirkpatrick framework) using the Professionalism Mini-evaluation Exercise (P-MEX). The content validity assessment showed that five items (items 4, 5, 12, 20, and 21) were not suitable for evaluating the TUMS clerks, and thus, were omitted. The internal consistency of the 16-item scale was found to be 0.95, based on Cronach's alpha. Since each item of the P-MEX is graded as follows: 1= unacceptable; 2= below expectations; 3= met expectations; and 4=exceeded expectations, the total score ranged from 16 to 64. Interns, residents, fellows, and faculty members used P-MEX to assess participants. One of the researchers provided the necessary instructions on the tool before conducting the assessments. Data analysis was carried out only for students whose evaluations were completed before and after the intervention, and for those who participated in three sessions and more.

***Statistics***

The demographics of participants were summarized using frequency, mean, and standard deviation. The paired t-test was performed to compare means of SJT, QUBPI, and P-MEX scores. Multiple Linear Regression (MLR) was performed using medical students' post-test mean scores of SJT, QUBPI, and P-MEX as the dependent variables; and the pre-test mean scores and the group as the independent variables. All data analyses were performed using SPSS version 21(23).

The study protocol was approved by the Ethics Committee of Tehran University of Medical Sciences (TUMS) (No. IR.TUMS.MEDICINE.REC.1395.1722).

## Results

Among 105 eligible students, 75 (71.4%) voluntarily participated in this study and were randomly allocated to the intervention and control groups. The mean age of students in the intervention group was 22.12 years (SD=0.95), and 44.7% (N=17) were female. The mean age of the control group was 22.77 years (SD=0.95), and 35.5% (N=11) were female. There was no significant difference between the mean age of the students in the control and intervention groups. 

The mean and standard deviation of pretest and posttest scores of judgment (SJT), Attitude (QUBPI), and behavior (P-MEX), before and after the program is presented for the control and intervention groups in Table 1.

**Table 1:** Results of paired t-test analyses to compare mean scores of SJT, QUBPI, and P-MEX in the intervention and control groups, before and after the educational program

Test (Outcome)	Group	Pre-test				Post-test			
		Mean	Standard Deviation	t	Sig.	Mean	Standard Deviation	t	Sig.
Situational Judgment Test (Cognitive base)	*Intervention * *(n=31)*	7.56	2.49	-1.60	0.11	10.17	2.17	4.81*	<0.001
	*Control * *(n=28)*	8.57	2.40			7.71	1.74		
Queen University Belfast Professionalism Inventory (Attitude)	*Intervention * *(n=36)*	56.72	5.48	0.14	0.89	59.17	6.65	0.97	0.33
	*Control * *(n=31)*	56.48	8.34			57.10	10.08		
Professionalism Mini Evaluation Exercise (Behavior)	*Intervention * *(n=25)*	47.42	4.17	-0.53	0.59	48.85	5.51	2.24**	0.03
	*Control * *(n=25)*	48.07	4.36			45.74	4.18		

* *Correlation is significant at the level of 0.05.*

** *Correlation is significant at the level of 0.01.*

Post-test judgment was improved in the intervention group, showing a significant difference between the control and intervention groups (*P*<0.001). Based on the results of the MLR of SJT, independent variables of “group” and “mean pre-test score” explained 64% (R^2^) of variance scores (dependent variable). Considering the students' attitude (QUBPI), the MLR showed that independent variables of "group" and "mean pre-test score" explained only 16% of the variance in the mean score. The MLR of students' behaviors (P-MEX) showed that independent variables of "group" and "pre-test behavior score" could explain only 0.23% of the variance in the mean score. 

It should be noted that R^2^ must be somewhere in the range of 0 and 1, where 0 shows that the result can't be anticipated by any of the independent variables and 1 demonstrates that the result can be anticipated without error from the independent variables. Based on the results of the MLR of students' judgment, by a one-unit increase in the mean pre-test score, the mean score of the post-test increased by 0.66 units (*P*< 0.001). Also, in line with the paired t-test results, after controlling the confounders using MLR, the students' judgment in the intervention group increased significantly in comparison with the control group (*P*< 0.001). Based on the results of the MLR, by a one-unit increase in the mean pre-test score of the students' attitudes, the mean score of post-test increased by 0.46 units (*P*< 0.001). However, after controlling the confounders using MLR, in line with the paired t-test results, there was no statistically significant difference between the two groups’ attitudes after the intervention (*P*= 0.34) (Table 2). 

**Table 2:** Results of paired t-test analyses to compare mean scores of SJT, QUBPI, and P-MEX in the intervention and control groups, before and after the educational program.

Test (Outcome)		Unstandardized Coefficients		Standardized Coefficients Beta	Sig.	R^2^
		B	SE			
Situational Judgment Test (Cognitive base)	*(constant)*	10.40	0.96		<0.001	0.64
	*group*	-2.80	0.48	- 0.47	<0.001	
	*Pre-test * *score*	0.66	0.09	0.55	<0.001	
Queen University Belfast Professionalism Inventory (Attitude)	*(constant)*	1.76	0.48		<0.001	0.16
	*group*	-0.10	0.11	-0.11	0.341	
	*Pre-test * *score*	0.46	0.13	0.39	<0.001	
Professionalism-Mini Evaluation Exercise (Behavior)	*(constant)*	2.92	0.71		<0.001	0.23
	*group*	-0.17	0.09	-0.23	0.074	
	*Pre-test * *score*	0.77	0.23	0.43	<0.001	

In the intervention group, in comparison with the control group, the mean score of students' behavior increased by 0.17 units, provided that the variable pre-test value was considered constant (*P*= 0.07). Also, with a one-unit increase in the mean pre-test score of students' behavior, the mean score of the post-test increased by 0.77 units (*P*< 0.001). However, after controlling the confounders using MLR in line with the paired t-test results, professional behavior in the intervention group did not show any statistically significant difference (*P*= 0.07).

## Discussion

To improve the cognitive base, attitude, and professional behavior of medical students, we conducted a pre-test post-test randomized controlled study by designing and implementing a 10-hour program through 16 weeks, based on the Holmes' reflection approach. We found that the students' judgment on professionalism improved in the intervention group compared to their baseline scores and compared to the control group; however, there were no significant difference in the students' attitude or performance.

There are few studies that have tried to investigate the impact of eliciting reflection as a method of professionalism training. Holtman et al. evaluated the effectiveness of a professionalism course in an academic plastic using a pre- and post-tests study design. They implemented a 12-hour course through 6-week for health care professionals in plastic surgery (faculty members, residents, nurses, and medical students). Teaching methods included didactic lectures, journal clubs, small group discussions, and book reviews. They assessed the outcomes using four levels of the Kirkpatrick model. Given the qualitative nature of the data extracted from levels 3 and 4 in this study, outcomes were measured by the incidence of sentinel events. The results showed improvement in knowledge and professional behaviors. In addition, the participants were satisfied with the course; however, their attitude toward professionalism did not change. Since the authors did not use a randomized design, it is difficult to attribute all the changes to the training program. In addition, participants of the study included a diverse range of health professionals who were more involved in patient care, compared to the clerks in our study (24). 

Given the lack of comparable quantitative, randomized studies on teaching and assessing professionalism, our ﬁndings can be considered in a wider context including medical ethics and ethical judgement.

Murrell conducted a cross-sectional study to evaluate medical students' ethical judgment. The results showed that there was no significant difference between students who participated in the professionalism course and those who did not (25). This finding is consistent with the findings of our study in which confounding factors in the clinical setting as well as the short duration of study might have caused the absence of any difference in the scores. Moreover, Goldie et al. in a quasi-experimental study evaluated the effectiveness of teaching ethics in small groups. Training had a significant positive impact on the students' potential ethical behaviors. It also created a supportive environment for promoting constructive criticism of peers' performance. In this study, again, the intervention group attitude towards professionalism did not differ significantly compared to the control group (26), which is consistent with the present findings.

Clearly, there are many variables that could have influenced the professional behaviors of learners and their attitudes toward professionalism. Hence, we were not able to detect a significant improvement in these two outcomes. The hidden curriculum is a very powerful tool for encouraging professional values (7, 27); yet, it “also has a more pernicious side, whereby professionalism lapses and unethical behaviors are normalized, particularly in the clinical setting” (28). Students work in the clinical settings under the supervision of attending physicians and residents and as the lowest-ranking members of the clinical chain, they are subject to implicit learning (25). According to Coulehan and Williams "the tacit socialization process is powerful because it is continuous throughout the clinical training and is reinforced by doing rather than saying" (29). Based on the results of previous studies, conducted in hospitals affiliated to TUMS, it can be concluded that inappropriate or negative role models may contribute to the inefficacy of the training programs that aim to develop professionalism among students (27, 30). Having said that, there might also be other reasons for the lack of significant behavioral differences after the intervention in our study. First, the P-MEX might have not been the perfect assessment tool for our study, despite its previous validations and despite our modifications (31). 

To the best of our knowledge, this is the first true experimental research that evaluated a professionalism training program based on a guided reflection method. The current study had several strengths. First of all, we used a conceptual framework that facilitated guided reflection and feedback in multiple sessions. Interestingly, while the Holmes' approach is grounded in a theoretical basis, it is very practical and applicable to the real life. To make the most out of this approach, we managed to design our intervention as a longitudinal program, because standalone educational courses are considered to be less effective. Besides, to provide robust evidence on the effects of our program, we included a control group and performed pre-post comparisons. We also used previously established instruments for all of our measurements, including the cognitive base and attitude of students, as well as their actual behavior, an outcome that has often been ignored in previous studies. 

On the other hand, our study had also a few limitations. Despite orientating the intervention group not to share material with the control group, information exchange was possible between the two groups. In addition, while we tried to minimize the differences between small group facilitators, through training and written guidelines, their diverse experiences might have resulted in different discussions that in turn could have caused inconsistency in our program. As the TUMS clerks are not involved in direct patient care, so direct observation of their performance using P-MEX might have not been always possible. Secondly, in spite of our effort to train the raters, it is possible that some of residents, fellows, or faculty members have failed to contribute to a valid assessment due to their high workload.

Finally, our study was performed on a small scale, we did not evaluate the longterm outcomes, and we did not collect qualitative data which could have provided us with a deep understanding about the impact of the program.

## Conclusion

The application of Holmes’ reflection approach improved the judgment of professionalism among medical students upon their transition to the clinical settings. It is recommended to conduct mixed-methods studies with long-term follow-ups to better evaluate the effects of reflection on the attitudes and professional behaviors of students. 

## Notes:

**Citation to this article:**

Naeimi L, Asghari F, Nedjat S, Mirzazadeh A, Abbaszadeh M, Sima AR, Mortaz Hejri S. Turning unprofessional behaviors around using Holmes' reflection approach: a randomized controlled study. J Med Ethics Hist Med. 2020; 13: 12.
